# Assessment of lipid profile and its association with acne vulgaris severity in adolescents and young adults: A cross-sectional study in Kurdistan Region, Iraq

**DOI:** 10.5339/qmj.2025.10

**Published:** 2025-02-23

**Authors:** Azzam Abdulsattar Mosa, Mohammad Ahmad Hamza, Mohammed Yaseen Khalaf

**Affiliations:** ^1^Department of Chemistry, College of Science, University of Duhok, Kurdistan Region, Iraq; ^2^Department of Chemistry, College of science, University of Zakho, Kurdistan Region, Iraq *Correspondence: Mohammad Ahmad Hamza. Email: mohammad.hamza@uoz.edu.krd

**Keywords:** Acne, apolipoprotein A, cholesterol, triglycerides, HDL-cholesterol

## Abstract

**Background:**

The relationship between acne vulgaris and lipid profiles has been the subject of limited research across diverse populations, yielding conflicting results. The aim of this study was to determine whether there are any significant differences in lipid profile and selected apolipoproteins between two groups: adolescents and young adults with acne vulgaris and an age- and sex-matched control group. Additionally, the study aimed to identify indicators associated with severe acne vulgaris.

**Methods:**

The cross-sectional study involved 100 adolescent and young adult patients (50 adolescents aged 11–18 years and 50 young adults aged 19–26 years) who were visitors to the Dermatology Unit of Azadi Teaching Hospital in Duhok City, Kurdistan Region of Iraq, diagnosed with acne vulgaris. These patients were compared with a control group of 90 healthy individuals who were matched for age, sex, and BMI (body mass index). Measurements included lipid profile, apolipoprotein A (Apo A), apolipoprotein B, and lipase. The Ethics Committee of the Directorate of Health of Duhok City Governate approved the study (reference number: 15092021-9-3). Data were statistically analyzed using SPSS software (version 26.0 for Windows), and the *p* value ≤ 0.05 was considered statistically significant.

**Results:**

The results showed an increase in total cholesterol, triglycerides, HDL-C (high-density lipoprotein cholesterol), non-HDL-C, and Apo A in the acne vulgaris group compared with the control group (157.5 ± 36, 125.4 ± 50.5, 40.9 ± 10.9, 114.6 ± 41, and 189.5 ± 26 versus 129 ± 22.5, 98.1 ± 49.9, 33.4 ± 8.1, 95.6 ± 32.1 and 179.6 ± 22.4, respectively), all with *p* values of ≤ 0.05. The regression model showed that an increase in one unit of cholesterol resulted in a 4% increase in the odds of acne vulgaris (*p* < 0.001). The severity of acne vulgaris was associated with age (mild (17.9 ± 2.7), moderate (18.09 ± 2.8), severe (20 ± 2.7), *p* < 0.05) and with a decrease in Apo A levels compared with the mild group (179.2 ± 25.5 and 200 ± 25.9, respectively, *p* < 0.05). There was a significant increase in non-HDL-C levels in young adult patients compared with adolescent patients (125.8 ± 40.3 versus 103.5 ± 39.9, *p* = 0.01).

**Conclusions:**

High cholesterol is a feature of adolescent and young adult patients with acne vulgaris. Older patients tend to have more severe forms of acne, which are significantly associated with elevated non-HDL-C levels. Decreased Apo A levels have also been identified as an additional indicator of severe cases of acne vulgaris.

## Introduction

Acne vulgaris is prevalent among adolescents and young adults worldwide, increasing the burden of treatment, education, and psychosocial support. According to the latest statistics, the incidence of acne has risen by approximately 0.55% annually between 1990 and 2019.^1^ Hormonal changes in adolescence, diet, and oxidative stress are major factors that play a vital role in the development of acne. In addition, *Propionibacterium acnes*, immunodeficiency, unhealthy habits, and lack of education can exacerbate acne.^2,3^

It is well known that a high-carbohydrate diet is associated with increased levels of cholesterol and triglycerides in all lipoproteins, which has been confirmed by the latest investigations.^4,5^ In addition, there is solid evidence that a high-glycemic diet is associated with the occurrence of acne vulgaris. Conversely, a low-carbohydrate diet with the use of omega-3 supplements could be effective in reducing acne lesions.^6,7^

It appears that lipids may play a role in the proliferation of acne, although the exact mechanism remains elusive. The association between acne vulgaris and lipid profiles has been the subject of limited research across various ethnic populations, leading to inconsistent findings. In one study, elevated cholesterol levels were found in 45 Iranian patients with acne vulgaris of both genders, compared to healthy controls, with no significant differences in LDL, HDL, and triglyceride levels.^8^ Conversely, lipid profile levels were higher in 100 Saudi patients with acne vulgaris than in their 104 otherwise healthy controls.^9^ In Chinese individuals, researchers also found abnormally high lipid profile levels in 181 acne vulgaris patients compared with 131 age- and sex-matched healthy controls.^10^ Furthermore, a separate study found significantly higher triglyceride and LDL levels in female patients with acne vulgaris, but not in male patients in the Iraqi population.^11^ In contrast, lipid levels in 30 Indonesian patients with acne vulgaris did not show significant differences compared to their 30 controls.^12^ The discrepancy in the above results could be due to several factors such as small sample size,^8,12^ dietary and lifestyle routines, as well as ethnic and cultural differences.^2,13^

Apolipoproteins also known as apoproteins, protein components of lipoproteins, play a crucial role in their metabolism and transport. Apolipoprotein A is an important component of HDL and has anti-inflammatory properties.^14^ Researchers have linked low levels of apolipoprotein A in patients with severe cystic acne to an increased risk of atherosclerosis due to the inverse correlation between apolipoprotein A and atherosclerosis.^15^ Apolipoprotein B has pro-inflammatory properties and occurs in the LDL and very-low-density lipoprotein (VLDL) particles.^14^ A recent study found that reducing LDL and VLDL particles rich in apolipoprotein B by lipid-lowering drugs could reduce the risk of acne vulgaris.^16^ Detecting potential changes in apolipoprotein levels in people with acne vulgaris could fill gaps in our knowledge of the disease mechanism. The aforementioned factors demand a comprehensive investigation into the correlation between lipids and acne vulgaris, considering parameters such as gender, age, lipase, and apolipoproteins. Moreover, there is no previous work on the association of lipid profiles with acne vulgaris in the Kurdish population.

The aim of the present cross-sectional study was to determine whether there are significant differences in the levels of lipid profile, lipase, and the selected apolipoproteins between two groups: adolescents and young adults with acne vulgaris and an age- and sex-matched control group. The study also aimed to identify a potential indicator for the progression of the disorder to severe stages using a multiple-comparison approach among patients with mild, moderate, and severe acne vulgaris.

## Methods

### Study design

In the present cross-sectional study, we examined a cohort of 190 participants, consisting of adolescents and young adults of both genders. G-Power software was used for a prior power analysis calculation to estimate the appropriate sample size of the study. This gave an expected effect size of 0.5, a two-tailed test, an α error probability (err prob) of 0.05, and a power (1 − β err prob) of about 0.93. A consecutive sampling method was used to enroll patients who met the inclusion criteria. Acne vulgaris was diagnosed in 100 patients (50 adolescents aged 11–18 years and 50 young adults aged 19–26 years). These patients were among the visitors to the Dermatology Unit of Azadi Teaching Hospital in Duhok City, in the Kurdistan Region of Iraq. The mean and standard deviation (mean ± SD) of age for this group was 18.5 ± 2.8.

**Figure 1.**
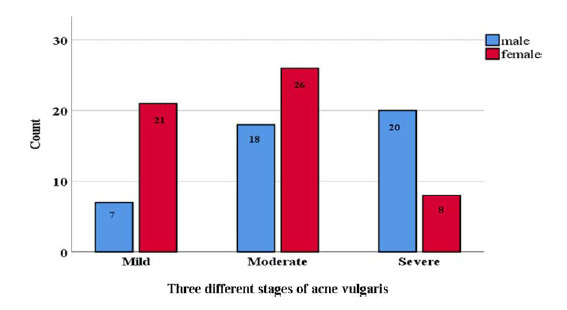
Distribution of male and female patients with different stages of acne vulgaris according to the global acne grading system.

A convenience sampling approach was used for the control group. A total of 90 healthy individuals (32 adolescents aged 11–18 years and 58 young adults aged 19–26 years) remained after excluding 10 individuals who did not meet the BMI (body mass index) of the patient group. These participants were carefully selected to match the first group in terms of age, sex, and BMI distribution to ensure comparability with the patient group. They were selected from secondary schools and university students. The mean and SD of age for the control group was 18.9 ± 2.9. All participants underwent a thorough examination by dermatologists. Patients of both genders were sub-classified into 28 mild, 44 moderate, and 28 severe groups depending on the severity of acne vulgaris according to the global acne grading system, as shown in Figure 1.^17^

### Inclusion criteria

Inclusion criteria were adolescent and young adult patients (aged 11–26 years) of both genders with different stages of acne vulgaris and an age-, sex-, and BMI-matched control group without acne vulgaris.

### Exclusion criteria

Exclusion criteria were cases taking medical therapy such as statins and hormonal therapy, as well as cases with abnormally high BMI, pregnancy, dyslipidemia, and metabolic syndrome. A questionnaire was completed for patients and controls, which included the following: name, age, gender, height, weight, type of acne, education, illiteracy, marital status, chronic disease, treatment, and family history. Informed consent was obtained from each participant before the start of the study. The study was approved by the Ethics Committee of the Directorate of Health of Duhok City Governate (reference number: 15092021-9-3).

### Sample collection and processing

After 12 hours of fasting, 5 ml of venous blood was collected from each participant and transferred to gel tubes. Sera were obtained after centrifugation at 3,500 rpm for 10 min and stored at -70^°^C until used for biochemical measurements.

### Biochemical measurements

Estimation of the levels of total cholesterol (TC), triglycerides (TG), low-density lipoprotein cholesterol (LDL-C), high-density lipoprotein cholesterol (HDL-C), apolipoprotein A (Apo A), apolipoprotein B (Apo B), and lipase were carried out in the laboratory of Vin Private Hospital, Duhok by using commercially available kits (Roche Diagnostics, Mannheim, Germany). Analyses were performed according to the manufacturer's instructions using a COBAS 6000 Hitachi Automatic Analyzer. The non-HDL-C is equal to TC minus HDL-C (non-HDL-C = TC - HDL-C). BMI is weight (kg) divided by the square of height (m^2^) (BMI = weight (kg)/height (m^2^)).

### Statistical analysis

Data normality was tested using the Shapiro–Wilk test, and all data are presented as mean ± standard deviation (SD) for consistency. Independent-samples t-test, Mann–Whitney test, and chi-square test were used for comparison between two groups. Multiple logistic regression was used to evaluate the association between variables and acne vulgaris. ANOVA and Kruskal–Wallis tests were used to determine the significant differences between three groups. SPSS software (version 26.0 for Windows) was used, and a value of *p* ≤ 0.05 was considered statistically significant.

## Results

The comparison between the acne vulgaris patient and control groups showed an increase in TC, TG, HDL-C, non-HDL-C, and Apo A in the group with acne vulgaris compared with the control group. These differences were apparent in both genders, except Apo A levels, which increased significantly in females but not in males. No significant difference was found in LDL-C levels between patients and controls, but a significant increase was observed in female patients compared with female controls. There was also no significant difference in lipase activity levels between patients and controls, but a significant decrease was noted in male patients compared with controls, as shown in Table 1.

On the one hand, the variables that showed a significant difference between patients and controls in Table 1 were selected. TG and non-HDL-C were excluded because they did not meet the multiple logistic regression assumption. The dependent categorical variables were “patients” and “controls”. Cholesterol, HDL-C, Apo A, and gender were used as independent variables. As shown in Table 2, the regression model indicated that an increase in one unit of cholesterol increased the odds of acne vulgaris by 4% (*p* < 0.001). An increase in one unit of HDL-C increased the odds of acne vulgaris by 10.4% (*p* < 0.001). The Nagelkerke *R* ^2^ was 43.3, and the percentage of prediction was 75.3%. The differences in gender and Apo A levels were not significant.

The comparison of variables among the three groups of acne vulgaris showed that age was a significant factor in the development of acne vulgaris, as shown in Table 3. There was a significant increase in age in the severe group compared with the mild and moderate groups. In addition, there was a significant decrease in Apo A levels in the severe group compared with the mild group. No significant difference was observed in the other variables.

**Figure 2.**
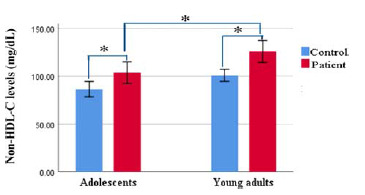
Comparison of non-HDL-C levels by age group. A significant increase in non-HDL-C levels was observed in young adult patients compared to adolescent patients (*p < 0.05).

The comparison between adolescent patients and young adult patients showed no significant differences between all variables, except non-HDL-C, which was significantly increased in young adult patients compared with adolescent patients (125.8 ± 40.3 versus 103.5 ± 39.9, *p* = 0.01), as shown in Figure 2.

## Discussion

Acne vulgaris is a chronic inflammatory disease that primarily affects the pilosebaceous unit and the sebaceous gland. This condition can occur in different parts of the body, affect different demographic groups, and result in a variety of skin lesions. Although numerous factors have been associated with the prevalence of acne vulgaris, the precise pathophysiology and primary cause of the disease remain to be fully elucidated.^18,19,20^ Ongoing research is focused on elucidating these aspects to enhance therapeutic strategies.

The present study found significant increases in TC, TG, HDL-C, Apo A, and non-HDL-C in acne vulgaris patients compared with controls. Gender analysis revealed similar results for males and females, with slight differences in certain variables. The significantly elevated Apo A levels were primarily attributed to female patients, even though there was a slight increase in male patients. There was a non-significant decrease in lipase levels in patients compared to controls. However, a significant decrease in lipase levels was observed in male patients compared with their controls, but not in females. Additionally, there was an 11.44% increase in LDL-C levels in patients compared with controls, but this increase was not statistically significant. The LDL-C levels of female patients were significantly higher than those of their healthy female controls.

Several studies were consistent with the current results. Jiang et al. compared 90 females with severe acne vulgaris with 90 age-matched controls. They showed that TC, HDL-C, LDL-C, and Apo A significantly increased in patients.^10^ AbdElneam et al. found a significant increase in all lipid profiles in 100 acne vulgaris patients compared with controls.^9^ In 150 non-obese acne vulgaris patients, Manzoor et al. found a significant increase in the levels of TC, TG, VLDL, and the TG/HDL-C ratio compared with 80 age- and sex-matched controls.^21^ Furthermore, Hasrat and Al-Yassen found a significant increase in TG and LDL-C, but not in TC and HDL-C.^11^ In addition, Sobhan et al. showed that TC levels were significantly higher in 45 acne patients, with non-significant elevations in TG, HDL-C, and LDL-C levels. Multiple logistic regression showed a significant odds ratio for TC and LDL-C levels, but not for HDL-C and TG.^8^ However, the multiple logistic regression model of the present study showed that TC and HDL-C were associated with acne vulgaris.

Age and low Apo A levels appeared to be the characteristics of the severe cases compared with the mild and moderate groups. Apo A, the essential structural protein, is necessary for the function of HDL. Approximately 70% of HDL consists of this protein. It is crucial for reverse cholesterol transport, which enhances the cardiovascular system, boosts immunity during inflammation, and provides protection against viral or bacterial infections.^22^ Decreased levels in severe cases could be an indicator of acne vulgaris progression. These results need further confirmation and require future research.

In addition, there were no significant differences between the adolescent and young adult groups of patients except for a significant increase in non-HDL-C in the young adult group. However, it is evident that lipid metabolism is crucial for the progression of acne vulgaris, as shown by the results of both the present study and previous research. Several factors may be associated with acne progression, but the inflammatory response and abnormal immune cell infiltration could be considered as primary initiating factors, aggravated by abnormal lipid production. Dysfunction of lipid metabolism, which manifests itself in acne patients, can lead to greater inflammation or damage to the affected tissue by sending signals to pro-inflammatory cytokines and reprogramming the immune cells in acne lesions.^23^ In addition, the increased cholesterol levels in all types of lipoproteins in acne patients may be proportionally correlated with increased levels of free radicals. In fact, high-fat or high-carbohydrate diets have been shown to be associated with high levels of reactive oxygen species that initiate protein carbonylation and lipid peroxidation, as well as weaken the antioxidant defense system.^24^ A suppressed antioxidant defense system may allow acne bacteria to revive and increase their activity. Recently, a systematic review and meta-analysis suggested that oxidative stress may be present in acne vulgaris, and that the severity of acne vulgaris may also be correlated with oxidative stress.^25^ However, more research is important to prove the association of oxidative stress with the prevalence and progression of acne vulgaris.

In general, this study highlights a significant increase in lipid profile levels, particularly TC, in patients with acne vulgaris of both genders. Furthermore, it was noted that age and low Apo A levels were found to correlate with the progression of acne vulgaris to a severe condition. Additionally, a significant increase in non-HDL-C levels was observed in young adult patients compared with adolescent patients. Subsequent research is crucial to validate low Apo A levels as a potential biomarker for various skin disorders, including acne vulgaris.

## Conclusions

Elevated cholesterol levels are a clear feature in adolescents and young adults suffering from acne. This study showed increased cholesterol levels in all types of lipoproteins. Regression analysis showed that an increase of one unit in cholesterol levels increased the odds of developing acne vulgaris by 4%. Additionally, older patients tended to have more severe forms of acne, which were significantly associated with elevated non-HDL-C levels. Decreased Apo A levels were also identified as an additional indicator of severe acne cases. However, this result requires further confirmation by future research.

### Competing interests

The authors declare that they have no competing interests.

### List of abbreviations

[Table tbl4]

## Figures and Tables

**Table 1.** Anthropometric measurements and lipid profile, Apo A, and Apo B levels in the acne vulgaris patient and control groups.

Parameters	Patients (mean ± SD)	Controls (mean ± SD)	*p*

Number of participants	100	90	0.468^c^

Age (years)			

Overall Male Female	18.5 ± 2.8 18.8 ± 2.8 18.3 ± 2.9	18.9 ± 2.9 19.2 ± 2.6 18.6 ± 3.1	0.247^m^ 0.288^m^ 0.673^t^

Gender (*n*)			

Male Female	45 55	47 43	0.838^c^ 0.225^c^

BMI (kg/m^2^)			

Overall Male Female	20.7 ± 3.3 20.3 ± 3.3 21.07 ± 3.37	21.56 ± 2.78 21.2 ± 2.5 21.9 ± 2.99	0.064^t^ 0.136^t^ 0.193^t^

TC (mg/dL)			

Overall Male Female	157.5 ± 36 155.4 ± 34.8 159.3 ± 37.2	129 ± 22.5 129.3 ± 24.1 128.7 ± 20.9	8.8 × 10^-10^*^m^ 5.8 × 10^-5^*^m^ 7 × 10^-6^*^m^

TG (mg/dL)			

Overall Male Female	125.4 ± 50.5 143.5 ± 53.7 110.7 ± 42.8	98.1 ± 49.9 108.2 ± 53 87.1 ± 44.2	5 × 10^-6^*^m^ 2.8 × 10^-4^*^m^ 3.6 × 10^-4^*^m^

HDL-C (mg/dL)			

Overall Male Female	40.9 ± 10.9 40.2 ± 10.2 41.4 ± 11.5	33.4 ± 8.1 31.7 ± 7.5 35.18 ± 8.4	1.5 × 10^-7^*^m^ 2.7 × 10^-5^*^m^ 0.003*^t^

LDL-C (mg/dL)			

Overall Male Female	77.9 ± 27 74.3 ± 27.3 80.8 ± 27.1	69.9 ± 17.9 69.8 ± 20.8 70.1 ± 14.4	0.108^m^ 0.609^m^ 0.014*^t^

Apo A (mg/dL)			

Overall Male Female	189.5 ± 26 184.8 ± 22.4 193.3 ± 29.3	179.6 ± 22.4 180.2 ± 22.9 178.9 ± 22.1	0.009*^m^ 0.327^t^ 0.01*^m^

Apo B (mg/dL)			

Overall Male Female	76.8 ± 19.7 76.7 ± 20.2 76.9 ± 19.4	72.38 ± 13.3 72.8 ± 12.5 71.9 ± 14.3	0.071^m^ 0.329^t^ 0.149^m^

Lipase (U/L)			

Overall Male Female	21.9 ± 6.3 23.0 ± 6.2 21.1 ± 6.4	23.9 ± 21 26.1 ± 28.5 21.5 ± 8.1	0.174^m^ 0.043*^m^ 0.974^t^

Non-HDL-C (mg/dL)			

Overall Male Female	114.6 ± 41 110.7 ± 41 117.8 ± 41.2	95.6 ± 32.1 97.6 ± 25.6 93.5 ± 23.2	1.7 × 10^-4^*^m^ 0.033*^m^ 0.002*^m^


SD: standard deviation, BMI: body mass index, TC: total cholesterol, TG: triglyceride, HDL-C: high-density lipoprotein cholesterol, LDL-C: low-density lipoprotein cholesterol, Apo: apolipoprotein.

^c^Chi-square test, ^m^Mann–Whitney test, ^t^independent-sample t-test.

*The significant *p* value between patients and controls was set at ≤ 0.05.

**Table 2.** Multiple logistic regression model with the selected variables.

Variables	*B*	SE	Wald test	*p*	Exp(*B*)	95% CI	

						Lower	Upper

TC (mg/dL)	0.04	0.007	30.03	4.2 × 10^-8^*	1.04	1.026	1.055

HDL-C (mg/dL)	0.099	0.021	21.487	4 × 10^-6^*	1.104	1.059	1.151

Apo A (mg/dL)	0.013	0.008	2.699	0.1	1.013	0.998	1.028

Gender (female)	0.016	0.361	0.002	0.964	1.016	0.501	2.062

Constant	-11.53	2.091	30.43	0	0	-	-


The control group served as a reference.

*B*: unstandardized regression coefficient, SE: standard error, Exp(*B*): exponentiated *B*, CI: confidence interval, TC: total cholesterol, HDL-C: high-density lipoprotein cholesterol, Apo A: apolipoprotein A.

*The significant value was set at ≤ 0.05.

**Table 3.** Comparison of variables among the three groups of mild, moderate, and severe acne vulgaris according to the global acne grading system.

Parameters	Mild (*N*=28) (mean ± SD)	Moderate (*N*=44) (mean ± SD)	Severe (*N*=28) (mean ± SD)	*p*

Age (years)	17.9 ± 2.7	18.09 ± 2.8	20 ± 2.7^A,B^	0.015*^K^

BMI (kg/m^2^)	21.04 ± 3.4	20.9 ± 3.5	20.1 ± 3.0	0.51^V^

TC (mmol/L)	153.3 ± 33.9	163.2 ± 40	153.0 ± 28.9	0.638^K^

LDL-C (mmol/L)	77.3 ± 27.3	78.6 ± 27.3	77.4 ± 28.2	0.979^K^

HDL-C (mmol/L)	42.5 ± 9.2	41.8 ± 11.6	37.9 ± 11.1	0.239^V^

Triglyceride (mmol/L)	114.6 ± 44.6	128.4 ± 54	131.6 ± 50.5	0.421^K^

Apo A (mg/dL)	200 ± 25.9	189.4 ± 25.7	179.2 ± 25.5^A^	0.002*^K^

Apo B (mg/dL)	70.9 ± 16.5	79.9 ± 23.1	77.9 ± 15.9	0.157^V^

Lipase (U/L)	20.5 ± 5.6	22.5 ± 6.0	22.5 ± 7.4	0.259^K^

Non-HDL-C (mg/dL)	103.7 ± 44.0	121.4 ± 44.6	114.9 ± 31.5	0.32^K^


*N*: number, BMI: body mass index, TC: total cholesterol, LDL-C: low-density lipoprotein cholesterol, HDL-C: high-density lipoprotein cholesterol, Apo: apolipoprotein.

^K^Kruskal–Wallis test, ^v^ANOVA test.

^A^Significant difference between severe and mild. ^B^Significant difference between severe and moderate.

*The significant *p* value was set at ≤ 0.05.

**Table tbl4:** 

Apo A	Apolipoprotein A

Apo B	Apolipoprotein B

HDL-C	High-Density Lipoprotein Cholesterol

LDL-C	Low-Density Lipoprotein Cholesterol

TC	Total Cholesterol

TG	Triglycerides

